# Vertebral artery dissection in term pregnancy after cervical spine manipulation: a case report and review the literature

**DOI:** 10.1186/s13256-021-03090-z

**Published:** 2021-10-20

**Authors:** Francesca Monari, Stefano Busani, Maria Giovanna Imbrogno, Isabella Neri, Massimo Girardis, Annamaria Ghirardini, Francesca Cavalleri, Fabio Facchinetti

**Affiliations:** 1 Obstetrics and Gynecology Unit, Mother - Infant and Adult Department of Medical and Surgical Sciences, University Hospital Policlinico of Modena, Via del Pozzo 71, 41124 Modena, Italy; 2 Department of Anaesthesia and Intensive Care, University Hospital of Modena, Modena, Italy; 3 Department of Neuroradiology, University Hospital Policlinico of Modena, Via del Pozzo 71, 41124 Modena, Italy

**Keywords:** Vertebral artery dissection, Pregnancy, Vertebrobasilar ischemia, Cervical spine manipulation, Osteopathy

## Abstract

**Background:**

Vertebral artery dissection is an uncommon, but potentially fatal, vascular event. This case aimed to describe the pathogenesis and clinical presentation of vertebral artery dissection in a term pregnant patient. Moreover, we focused on the differential diagnosis, reviewing the available evidence.

**Case presentation:**

A 39-year-old Caucasian woman presented at 38 + 4 weeks of gestation with a short-term history of vertigo, nausea, and vomiting. Symptoms appeared a few days after cervical spine manipulation by an osteopathic specialist. Urgent magnetic resonance imaging of the head was obtained and revealed an ischemic lesion of the right posterolateral portion of the brain bulb. A subsequent computed tomography angiographic scan of the head and neck showed a right vertebral artery dissection. Based on the correlation of the neurological manifestations and imaging findings, a diagnosis of vertebral artery dissection was established. The patient started low-dose acetylsalicylic acid and prophylactic enoxaparin following an urgent cesarean section.

**Conclusion:**

Vertebral artery dissection is a rare but potential cause of neurologic impairments in pregnancy and during the postpartum period. It should be considered in the differential diagnosis for women who present with headache and/or vertigo. Women with a history of migraines, hypertension, or autoimmune disorders in pregnancy are at higher risk, as well as following cervical spine manipulations. Prompt diagnosis and management of vertebral artery dissection are essential to ensure favorable outcomes.

## Background

Vertebral arterial dissection (VAD) is a rare complication of pregnancy and puerperium. A data registry reported that 2.4% of symptomatic, spontaneous VADs occurred in the postpartum period [1]. Aortic, coronary, and cervical/vertebral artery dissection was reported to be associated with preeclampsia in the antenatal setting [1]. On the other hand, VAD incidence in hypertensive disorders of pregnancy is unknown, due to the paucity of reports documenting only adverse outcomes [1]. Hormonal and mechanical factors might increase the risk of VAD during pregnancy and puerperium [2]. Indeed, identified predisposing factors of VAD include intimal injury related to Valsalva maneuvers during labor, and alterations in arterial wall integrity due to hormonal or vasoactive substances, in addition to an overall state of hypercoagulability [3]. Another possible condition that can lead to VAD is cervical spine manipulation [4]. It is known that any type of trauma can cause a dissection such as cervical manipulation. Therefore, in nonpregnant patients, it is not uncommon, but most patients are asymptomatic, and this serious accident after manipulation has an underestimated incidence. Nevertheless, the risk related to the development of VAD is decidedly low if the manipulation maneuvers are carried out according to good clinical practice [4]. Several vascular and connective tissue disorders have also been associated with dissection; in particular, migraines, fibromuscular hyperplasia, and vascular Ehlers–Danlos syndrome [5]. As previously stated, the etiology of VAD is complex and often multifactorial, especially when trivial trauma and manipulations are involved. Other risk factors or conditions, such as fibromuscular dysplasia, Marfan’s syndrome, migraines, use of oral contraceptives, recent infections, and mild hyperhomocysteinemia, should be considered in any given case [6]. There exists uncertainty of how to counsel women with a previous VAD, regarding the risk of recurrence during pregnancy [2].

We aim to describe a 39-year-old female who presented with vertigo, nausea, and vomiting and was found to have a VAD. We discuss the presentation, differential diagnosis, and pathogenesis of this uncommon, but clinically significant, vascular event. Finally, we briefly review other described VAD cases.

## Case presentation

A 39-year-old pregnant Caucasian woman presented to the Obstetric Emergency Room reporting vertigo, vomiting, nystagmus, dizziness, and hindrance in the execution of fine movements of the right arm. The maternal parameters on admission are regular: pulse 98 beats per minute, pressure 110/68 mmHg, and temperature of 36.2 °C. She had an obstetric history of a first-trimester spontaneous abortion and a medical history of tension headache. She is married and graduated. She has a high socioeconomic status and is employed as an engineer.

The ongoing pregnancy coursed physiologically until that moment. The fetus was screened for aneuploidy with a noninvasive prenatal test (NIPT), while second- and third-trimester ultrasounds for the study of malformations were both normal. The oral glucose tolerance test at 24 weeks was negative. At 38 + 4-week gestation, the patient was hospitalized due to suspected vestibular neuritis diagnosed by an otolaryngology (ORL) specialist. Following diagnosis, the patient started therapy with corticosteroids, including prednisone 5 mg two times per day and levosulpiride 25 mg two times per day, that continued for 3 days.

On day 2, the patient developed diplopia and worsening of vomiting and dizziness, with improvement of symptoms in left lateral decubitus. The ORL revaluation excluded peripheral vestibulopathy and progressed to an urgent brain computed tomography (CT) scan without contrast that excluded ischemic or hemorrhagic brain lesions.

**Fig. 1:**
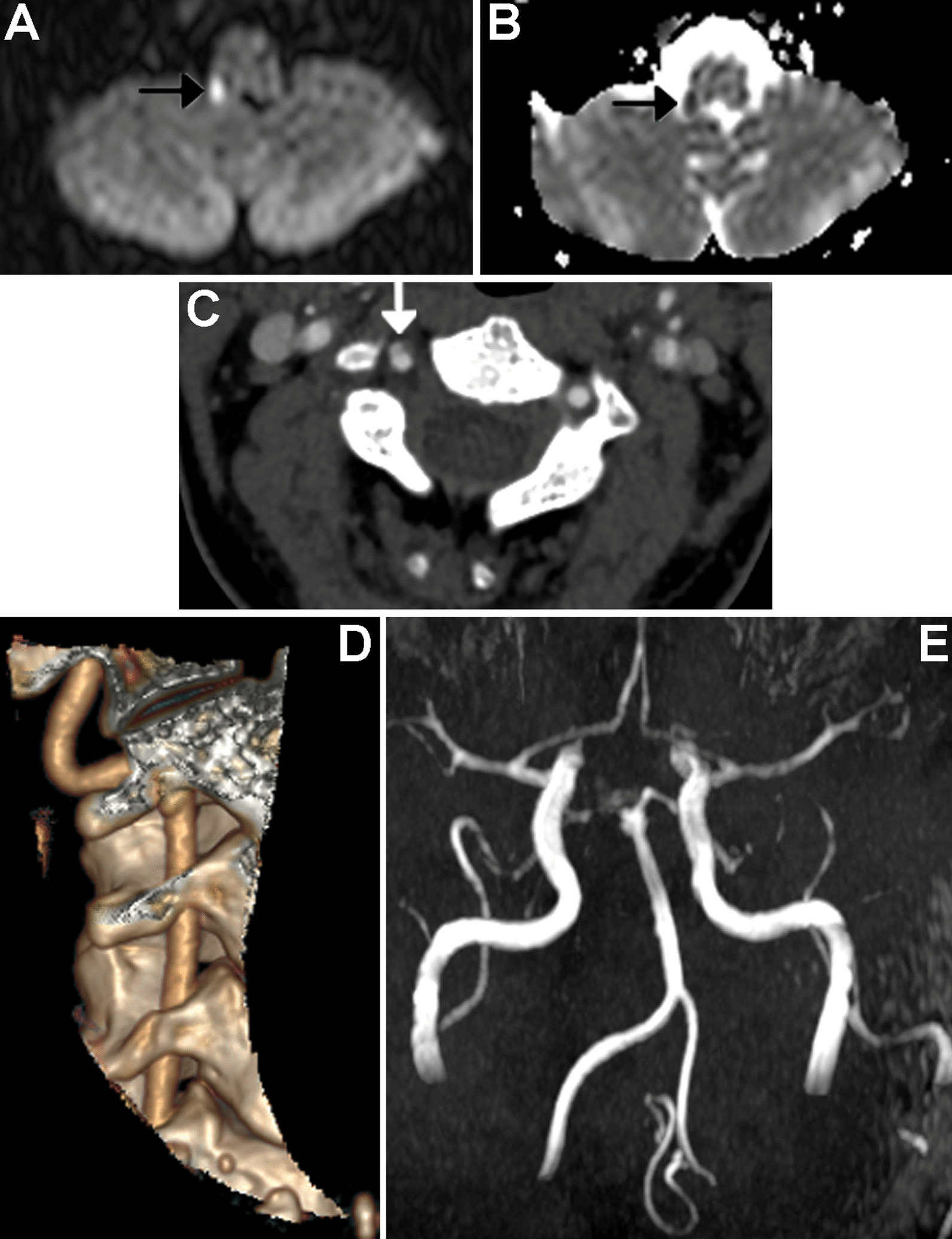
Right vertebral artery dissection with ischemia in the posterolateral medulla oblongata. In DWI (**a**) and ADC map (**b**) the arrow shows a punctate, shiny ischemic lesion, with typical reduction of ADC in the right posterolateral medulla oblongata.** c** and** d** CT angiography (axial and 3D reformat,** c** and** d**, respectively) showing a focal dissection of the V2 distal segment of the right vertebral artery, with the arrow in figure** c** pointing to the dissection.** e** MRI angiography (time of flight, TOF) showing the absence of visualization of right PICA

On day 3, due to further worsening of symptoms, urgent neurological counseling was performed. Viral examinations of herpes simplex I and II, herpes zoster, and herpes virus VI were negative. The neurologic examination showed the left eye adducted and elevated, vertical diplopia, and presence of rotatory nystagmus accentuated on the right gaze and dyssynergia in the cerebellar maneuvers of the right upper limb. After a detailed medical history, the patient stated for the first time that she had undergone cervical spine manipulations by an osteopathic specialist in the days preceding the beginning of the symptoms. Magnetic resonance imaging (MRI) of the brain was urgently performed, showing a punctate lesion hyperintense on diffusion-weighted imaging (DWI) (Fig. 1A–B), characterized by a reduction of apparent diffusion coefficient (ADC) on the colorimetric maps. This finding was suggestive of ischemia in the posterolateral right medulla oblongata, which is consistent with the symptoms of Wallenberg syndrome, although the patient did not have the full spectrum of symptoms. On MR angiography, the intracranial V4 segment was normal, but the right posteroinferior cerebellar artery (PICA) was not present (Fig. 1E). Urgent thrombolytic therapy or emergency revascularization was not deemed necessary by the neurologist colleague. Acetylsalicylic acid (ASA), 100 mg, therapy was then prescribed. Subsequently, echo-color Doppler ultrasound of the supra-aortic trunk detected no alterations of the cervical vessels, and transthoracic echocardiogram with exclusion of patency of the foramen ovale was performed. During this observation period, the patient was continuously monitored with noninvasive blood pressure monitor and pulse oximeter to detect hypotensive state and/or desaturation episodes early. Considering the clinical condition of the patient, the term gestational age, and initial onset of prodromal contractions, an elective cesarean section (CS) under subarachnoid anesthesia was performed, given the inability of the patient to deliver vaginally because of the ischemic cerebral event and obligatory left lateral decubitus, diplopia, and dizziness. The intraoperative and postoperative courses were uneventful. Six hours after the CS, prophylaxis with low-molecular-weight heparin was prescribed.

The neonate showed good adaptation to postnatal life with an Apgar score of 9 at 1′, 10 at 5′, and 10 at 10′; blood gas analysis was regular both in the artery and vein. Normal anthropometric parameters were present: 3250 g (52nd centile), length of 49 cm, and cranial circumference of 36 cm.

On day 4, for better study suspected dissection on small vertebral vessels, the patient underwent CT angiography of the neck, which showed a focal dissection at the V2 distal segment of the right vertebral artery (Fig. 1C–D). The puerperium course was normal, and the midwife helped the patient during breastfeeding because of the difficulty of standing up and walking due to the diplopic symptom. Psychological support was offered during the hospitalization, with daily physiotherapy rehabilitation and orthoptic evaluation. Congenital and acquired thrombophilia tested negative. After 12 days of rehabilitation, the patient was discharged with continued complaints of diplopia and a walker for mobility. After the VAD diagnosis and for the entire length of hospitalization, the patient was treated with Cardioaspirin 100 mg/day and prophylactic enoxaparin 4000 UI/day subcutaneous injection for 60 days. At the subsequent neurological evaluation, during the follow-up of 2 and 4 months, the patient showed persistence of vertical diplopia and a circumspect and wide gait, and life-long ASA was prescribed. A follow-up MRI was scheduled for 6 months after the stroke, which confirmed the signs of the previous ischemic lesion on the posterolateral right medulla oblongata. The remaining findings are unchanged.

Ethical approval was obtained, and the patient gave written informed consent to publish this case and any accompanying images.

## Discussion and conclusion

To our knowledge, this is one of the rare reports of an ischemic lesion due to VAD in low-risk pregnancy secondary to cervical spine manipulation. Cervical artery dissection (CAD), including VAD, is a rare complication of pregnancy; however, Salehi Omran *et al*. recently demonstrated that the incidence in pregnancy is twice as common as in the rest of the female population [7]. VAD has typically been associated with hypertensive disorder of pregnancy (HDP) [1, 8], autoimmune disease [9], and migraines [10]. A recent nationwide American cohort study on pregnancy-associated arterial dissection showed that VAD is the fourth most common dissection after or prior birth and found a significant association with older maternal age, chronic hypertension, dyslipidemia, tobacco use, alcohol use, obesity, heart failure, chronic liver disease, arthritis, depression, Marfan syndrome, and Ehlers–Danlos syndrome [11]. However, one recent meta-analysis demonstrated that nearly 50% of cases occur in the absence of such risk factors [12, 13]. To date, the etiology of VAD is not well established. Borelli *et al*. proposed a dual mechanism of pathogenesis occurring in the postpartum period: (1) advanced age, causing increased arterial stiffness, and (2) hormone fluctuations, inducing structural vascular changes [14], which may also happen at the end of pregnancy in our patient. McKinney *et al*. also suggested that endothelial damage may occur due to the release of vasoactive or angiogenic substances during pregnancy [15].

VAD should be considered in the differential diagnosis of women who present with nonspecific symptoms, such as headache, vomiting, and/or vertigo, particularly in the context of HDP [1]. Women older than 35 years and those with a history of HDP or autoimmune disease (that is, systemic lupus erythematosus, anti-phospholipid syndrome) are particularly at high risk. Other predisposing factors of arterial dissection in the peripartum period include intimal injury related to Valsalva maneuvers during labor, alterations in arterial wall integrity due to pregnancy-related hormonal or vasoactive substances [9], and reactive thrombocytosis (subsequent to postpartum hemorrhage), which may all play potential roles in this process and require further investigation [16]. Moreover, prompt diagnosis and management of VAD are essential to ensure favorable patient outcomes.

One recent report discussed the contributing factors in a case of VAD following chiropractic treatment in a pregnant woman with systemic lupus erythematosus [4]. Migraine disorder was shown to be associated with a twofold increased risk of VAD in a recent meta-analysis [9] and has been frequently reported in several case series [17–19]. Stuber *et al*. recently published a review of the literature regarding adverse effects of spinal manipulation in the pregnant and postpartum periods [20], identifying adverse events in five pregnant women and two postpartum women.

Table 1 summarizes all cases of VAD reported both prior and after delivery, with 24 cases distributed with a prevalence during the postpartum period (19 of the 24 cases). The clinical presentation is varied, with a higher frequency of headaches, vertigo, and diplopia, and the risk factors most represented are hypertension and migraines.

**Table 1:** Review of literature: cases of vertebral artery dissection VAD reported in pregnancy and postpartum period

Cases	Age	Presentation	Risk factors	VAD affected	Mode of delivery	Time to and from delivery (days)
Current report	39	Vertigo, vomiting, nystagmus, dizziness, and hindrance in the execution of fine movements with the right arm	Migraine	Right	Cesarean section	Antepartum, 39 w
Gasecki *et al*. (1999)	34	Neck pain, headache, 1 week later: right facial numbness, left-sided weakness, and vertigo, right. Horner’s syndrome, right-side ataxia,	Healthy	Right	Vaginal delivery	14 days postpartum
McKinney *et al*. (2002)	41	Severe headache, blurred vision, HTN	Preeclampsia	Left	Cesarean section	5 days postpartum
Tuluc (2006)	39	Headache preceding loss of consciousness	HTN	Right	Subsequent massive subarachnoid hemorrhage with death	Antepartum
Arnold *et al*. (2008)	41	Bilateral neck pain	Migraine, hyperlipidemia	Left	Vaginal delivery	18 days postpartum
	27	Ipsilateral neck pain, thunderclap headache	Migraine, HTN, hyperlipidemia	Right	Vaginal delivery	11 days postpartum
	38	Thunderclap headache	Migraine, hyperlipidemia	Bilateral	Vaginal delivery	7 days postpartum
	34	Ipsilateral neck pain, headache	Chiropractor neck manipulation	Right	Vaginal delivery	7 days postpartum
Sharma *et al*. (2010) [29]	28	Non-exertional, intermittent, substernal, sharp chest pain, and left arm numbness, intermittent bifrontal headache	Atherosclerotic risk factors	Left	Vaginal delivery	10 days postpartum
Cenkowski *et al*. (2012) [30]	35	Sudden-onset retrosternal chest pain radiating to the jaw, nausea, vomiting (7 months postpartum), diplopia, numbness to left arm and face (8 months postpartum)	None	Right	Vaginal delivery	7 months postpartum
Drazin *et al*. (2012)	37	Thunderclap headache, neck pain	Migraine, hHigh pressure during labor	Bilateral	Vaginal delivery	3 days postpartum
Morton A. (2012)	38	Occipital headache, severe right-sided anterior neck pain, ipsilateral Horner’s syndrome (after chiropractic treatment: spinal manipulation)	Migraine, SLE, HTN, heterozygous for prothrombin gene mutation	Right	4 days after the onset of neurological symptoms intrauterine fetal demise	Antepartum 16 weeks was noted
Kelly *et al*. (2014)	39	Thunderclap headache, ipsilateral neck pain, blurred vision, and horizontal diplopia	HTN, hyperlipidemia	Bilateral	Vaginal delivery	24 days postpartum
	39	Right eyelid ptosis, headache, and bilateral neck pain	Healthy	Right	Vaginal delivery	11 days postpartum
	29	Right-sided weakness, sensory loss, and expressive aphasia, followed by severe headache and right-sided hemiplegia	Migraine	Left	Vaginal delivery	53 days postpartum
	32	Severe headache and neck pain, followed by left-sided facial droop and left arm weakness	Migraine	Right	Vaginal delivery	0 days postpartum
	28	Severe headache, neck pain, bilateral leg weakness	HTN	Left	Cesarean section	4 days postpartum
Finley *et al*. (2015)	35	Thunderclap headache, intractable vertigo	Migraine	Right	Vaginal delivery	21 days postpartum
Nishimura *et al*. (2015)	35	Thunderclap headache	Eclampsia, PRES	Right		8 days postpartum
Shanmugalingam *et al*. (2016)	32	Left-sided neck pain	Preeclampsia/eclampsia	Left	Cesarean section	Antepartum, 38 + 2 weeks
	33	Right-sided neck pain	Preeclampsia	Right	Cesarean section	Antepartum, 36 weeks
	30	Headache with left-sided neck pain	NSAID-induced postpartum HTN, migraines, obesity	Right	Vaginal delivery	3 days postpartum
	30	Left-sided neck pain	Previous IUGR and postpartum hemorrhage with DIC	Left	Vaginal delivery	6 days postpartum,
Manasewitsch *et al*. (2020)	31	Frontal headache, vertigo, nausea, vomiting	Preeclampsia, smoking	Left	Cesarean section	10 days postpartum

*HTN* hypertension, *SLE* systemic lupus erythematosus, *PRES* posterior reversible encephalopathy syndrome, *NSAID* nonsteroidal antiinflammatory drug, *IUGR* intrauterine growth restriction, *DIC* disseminated intravasal coagulation

The association between cervical spine manipulation and neurovascular complications is still strongly debated [21, 22]. CAD is thought to occur spontaneously, but neck trauma, especially in hyperextension and rotation, has been reported as a trigger [23]. A population-based, case–control study found no evidence of excess risk of vertebrobasilar stroke associated with chiropractic care compared with controls [24]. A recent retrospective case–control study, however, found a significantly increased risk of VAD in individuals less than 55 years of age with recent neck manual therapy [25]. A recent multivariable regression analysis of a retrospective study assessed the risk factors and clinical outcomes associated with CAD-related strokes. Patients with CAD were younger and more likely to have a history of migraines and recent neck manipulation [26]. A systematic review and meta-analysis of chiropractic care and CAD concluded that the quality of the published data was very low, and the authors showed a small association between chiropractic neck manipulation and CAD [27, 28]*.*

Finally, the future of these women is somewhat debated. They should be advised about their increased risk of developing a new stroke, so these patients will need to continue Cardioaspirin prophylaxis for life [2]. Regarding reproductive future, a recent observational German study concluded that the risk of recurrent VAD may not be significantly increased with pregnancies, starting at least 12 months after the event, in women without connective tissue disease, such as our patient [2].

Despite the absence of hypertension and autoimmune diseases in our patient, previous chiropractic treatment, pregnancy hormonal condition, and advanced age (39 years) may have contributed to vessel fragility. The risk of VAD was also increased due to the history of tensive headache/migraine. Osteopathy practitioners should be aware of the possible complications of neck manipulation in pregnancy and the postpartum period, particularly in mothers with underlying medical disorders that may predispose to vessel fragility and VAD.

In conclusion, we recommend that obstetric professionals carefully consider VAD as a differential diagnosis when evaluating women with dizziness, headache, and neck pain with or without a recent history of spinal manipulation, both in pregnancy and in the postpartum period. Moreover, they should consider with caution the risks and benefits of any cervical osteopathy practice in pregnant women with risk factors for VAD (hypertension and autoimmune diseases, history of tensive headache/migraine).

## Data Availability

This is a case report of a single patient. To protect privacy and respect confidentiality, none of the raw data has been made available in any public repository. The original reports, laboratory studies, imaging studies, and outpatient clinic records are retained, as per normal procedure, within the medical records of our institution.

## Abbreviations

VAD: Vertebral artery dissection
CAD: Cervical artery dissection
NIPT: Noninvasive prenatal test
ORL: Otolaryngologist
CT: Computed tomography
MRI: Magnetic resonance imaging
ASA: Acetylsalicylic acid
CS: Cesarean section
HDP: Hypertensive disorder of pregnancy
DWI: Diffusion-weighted imaging
ADC: Apparent diffusion coefficient
PICA: Posteroinferior cerebellar artery
